# DEPTOR induces a partial epithelial-to-mesenchymal transition and metastasis via autocrine TGFβ1 signaling and is associated with poor prognosis in hepatocellular carcinoma

**DOI:** 10.1186/s13046-019-1220-1

**Published:** 2019-06-22

**Authors:** Jin Chen, Haidan Zhu, Qiumeng Liu, Deng Ning, Zhaoqi Zhang, Long Zhang, Jie Mo, Pengcheng Du, Xu Liu, Shasha Song, Yawei Fan, Huifang Liang, Jikui Liu, Bixiang Zhang, Xiaoping Chen

**Affiliations:** 1 Hepatic Surgery Center, Tongji Hospital, Tongji Medical College, Huazhong University of Science and Technology; Clinical Medicine Research Center for Hepatic Surgery of Hubei Province; Key Laboratory of Organ Transplantation, Ministry of Education and Ministry of Public Health, Wuhan, Hubei 430030 People’s Republic of China; 2 Department of Radiology, Tongji Hospital, Tongji Medical College, Huazhong University of Science and Technology (HUST), Wuhan, Hubei People’s Republic of China; 3 Department of Biliary and Pancreatic Surgery, Tongji Hospital, Tongji Medical College, Huazhong University of Science and Technology, Wuhan, 430030 People’s Republic of China; 4 Hepato-pancreato-biliary Surgery Department, Peking University Shenzhen Hospital, Shenzhen, Guangdong People’s Republic of China

**Keywords:** DEPTOR, Epithelial-to-mesenchymal transition, TGF-β, Snail, Hepatocellular carcinoma

## Abstract

**Background:**

DEPTOR is an endogenous inhibitor of mTORC1 and mTORC2 that plays a vital role in the progression of human malignances. However, the biological function of DEPTOR in HCC metastasis and the underlying molecular mechanisms are still unclear.

**Methods:**

Western blot analysis and immunohistochemistry(IHC) were employed to examine DEPTOR expression in HCC cell lines and tissues. A series of in vivo and in vitro assays were performed to determine the function of DEPTOR and the possible mechanisms underlying its role in HCC metastasis.

**Results:**

We found that DEPTOR was frequently overexpressed in HCC tissues, and its high expression was associated with high serum AFP levels, increased tumor size, vascular invasion and more advanced TMN and BCLC stage, as well as an overall poor prognosis. Functional experiments demonstrated that DEPTOR silencing inhibited the proliferation and mobility of HCC cells in vitro and suppressed tumor growth and metastasis of HCC cells in vivo. Accordingly, DEPTOR overexpression promoted the invasion and metastasis of HCC cells in vitro and in vivo, but had no effect on cell proliferation in vitro. Overexpression of DEPTOR induced EMT by snail induction. Conversely, knockdown of snail expression impaired the DEPTOR-induced migration, invasion and EMT of HCC cells. Furthermore, we found that the increase of snail expression by DEPTOR overexpression was due to an activation of TGF-β1-smad3/smad4 signaling possibly through feedback inhibition of mTOR.

**Conclusion:**

DEPTOR promotes the EMT and metastasis of HCC cells by activating the TGF-β1-smad3/smad4-snail pathway via mTOR inhibition. Therefore, targeting DEPTOR may be an ideal treatment strategy for inhibiting the growth and metastasis of HCC.

**Electronic supplementary material:**

The online version of this article (10.1186/s13046-019-1220-1) contains supplementary material, which is available to authorized users.

## Introduction

Hepatocellular carcinoma (HCC) is the sixth most common malignant tumor and the third leading cause of cancer-related mortality worldwide [1, 2]. Although surgical treatment is effective in removing localized HCC lesions [3], many patients still die from intrahepatic and extrahepatic metastases after curative resection [4, 5]. Therefore, there is an urgent need to uncover new molecular mechanisms underlying HCC metastasis, and thereby enable the development of new diagnostic and therapeutic strategies to prevent and treat metastases.

Epithelial-to-mesenchymal transition (EMT) plays a critical role in embryonic development, would healing, fibrosis and cancer metastasis [6]. EMT modifies the adhesion molecules expressed by the cell, which enhances the migration and invasion abilities of cancer cells. Cancer cells then disassociate from the primary carcinoma lesion and subsequently disseminate to distant sites [6]. Therefore, EMT is considered a key step of tumor metastasis [7]. EMT is driven by pleiotropic signaling factors such as EMT-inducing transcription factors (EMT-TFs: snail, slug, ZEB1, ZEB2, twist etc.), miRNAs and epigenetic and post-translational regulators [6, 8]. The loss of E-cadherin (encoded by CDH1) is one of the most important hallmarks of EMT, and was demonstrated to be essential for tumor invasion [9, 10]. Snail is a transcriptional repressor of E-cadherin that directly interacts with its promoter to inhibit transcription [11].

The role of TGF-β signaling in cancer is context-dependent [12, 13]. In premalignant lesion, TGF-β functions as a tumor suppressor by inducing cytostasis, differentiation or apoptosis of cancer cells [12, 14]. However, with tumor progression to malignancy, TGF-β signaling acquires a tumor-promoting function, promoting tumor growth and invasion, facilitating the evasion of immune surveillance, as well as cancer cell dissemination and metastasis [12, 13]. TGF-β signaling mainly consists of the canonical Smad pathway and the non-canonical, or Smad-independent pathway (e.g. ERK, P38, JNK) [12]. TGF-β enhances the migratory and invasive properties of cancer cells by inducing EMT [15]. TGF-β downregulates the expression of E-cadherin by upregulating snail through the Smad-dependent or independent pathway [15–17].

The DEP domain containing mTOR interacting (DEPTOR) protein was first identified as a binding partner of mTOR that inhibits the activity of mTORC1 and mTORC2 [18]. In addition to acting as an inhibitor of mTOR, which is hyperactivated in the majority of human cancers, DEPTOR may also act as an oncogene in certain cancers. DEPTOR is overexpressed and promotes cancer cell proliferation and survival by feedback activation of the PI3K/AKT pathway in various cancers including cervical squamous cell carcinoma, osteosarcoma, breast cancer, colorectal cancer and HCC [19–23]. However, the relationship between DEPTOR and metastasis has only been reported in breast cancer [22]. DEPTOR promotes the metastasis of triple-negative breast cancer in vivo by upregulating the expression of survivin [22]. However, whether DEPTOR promotes HCC metastasis is largely unknow.

In this study, we investigated the expression pattern of DEPTOR in human HCC and present evidence of its clinical significance. The tumorigenic and metastatic roles of DEPTOR in the development of HCC both in vivo and in vitro, and the underlying mechanisms by which it drives metastasis are further investigated.

## Materials and methods

### Patients and HCC tissue specimens

A total of 53 paired specimens of tumor and adjacent non-tumor tissues were collected from 53 HCC patients (45 men and 8 women, median age 47 years; age range 26–79 years) who underwent hepatectomy at the Hepatic Surgery Center, Tongji Hospital of Huazhong University of Science and Technology (HUST) (Wuhan, China). Matched fresh specimens of HCC tissues and adjacent non-tumorous liver tissues were lysed separately for western blot analysis. A tissue microarray of 110 pairs of primary HCC tissues as well as the clinical and prognostic data were acquired from the specimen library of the Hepatic Surgery Center, Tongji Hospital. The surgery dates ranged from Feb 16. 2012 to Mar 29. 2016, and the end point of the follow-up was June 2018. Overall survival (OS) was defined as the interval between the date of resection and the date of death or last follow-up. For surviving patients, the data were censored at the last follow-up.

### Plasmids, lentivirus, clone selection and RNA interference

The pLKO.1 - TRC cloning vector (plasmid #10878; Addgene, Cambridge, MA, USA) and pBABE-puro (plasmid # 1764; Addgene, Cambridge, MA, USA) were purchased from Addgene. The pMD2.G, gagpol and psPAX2 plasmids were a gift from Didier Trono (Addgene plasmids # 12259, #35614 and 12,260). Full-length human DEPTOR cDNA was amplified by PCR and subcloned into the lentiviral vector pBABE-puro to establish Bel-7402 and HepG2 cell lines that stably overexpress DEPTOR.

The DEPTOR shRNA oligos were purchased from GeneChem Co, Ltd., (Shanghai, China). Three DNA oligos were subcloned into the lentiviral vector pLKO.1 - TRC cloning vector to establish stable HLF and SMMC-7721 cell lines with DEPTOR knockdown. The nonspecific control target sequence was TTCTCCGAACGTGTCACGT. To obtain stable cell lines, HCC cells were infected with lentivirus for 24 h and selected in growth medium containing 5 μg/ml puromycin for 7 days. Stably transfected clones were validated by western blot analysis.

RNA interference was used to knock down Snail, Smad2 and Smad3. HCC cells were seeded into each well of a 6-well plate. Next day, the cells in each well were transfected with 5 μl of siRNA oligo (20 μM) plus 3.75 μl Lipofectamine 3000 for 48 or 72 h. Cells were collected to validate the knockdown efficiency. The target sequences of Snail shRNA#1 and #2 were AACTGCAAATAC TGCAACA and ACTCAGATGTCAAGAAGTA, respectively. The target sequences of shRNA and siRNA are listed in Additional file 2: Table S2.

#### Cell lines and reagents

MHCC-97H and HCC-LM3 cells were purchased from the Liver Cancer Institute of Fudan University, Shanghai, China. Huh7, Hep3B and HepG2 cells were purchased from the China Center for Type Culture Collection (CCTCC, Wuhan, China). The cells were cultured in Dulbecco’s modified Eagle’s medium (Invitrogen Corporation, Carlsbad, CA, USA) supplemented with 10% fetal bovine serum (Life Technologies Inc., Gibco/Brl Division, Grand Island, NY, USA) in a humidified atmosphere comprising 5% CO_2_ at 37 °C. The HepG2 and Hep3B cell lines were authenticated by comparative genomic array hybridization or short tandem repeat DNA profiling according to the ATCC database, which was performed within less than 10 passages after authentication and less than 20 passages after receipt from commercial suppliers. The other cell lines were identified using the STR genotyping test method by Wuhan Genecreate Biological engineering Co., Ltd., China.

Puromycin, everolimus, rapamycin and LY364947 (a TβR1 inhibitor) were purchased from Cayman Chemical Company (Ann Arbor, MI, USA).

### Luciferase reporter assay

The luciferase reporter assay was performed as described previously [24, 25]. Plasmids PGL4.17 and PGL4.48 were purchased from Promega Corporation (USA). Briefly, 1 × 10^5^ of the indicated cells per well were seeded into 24-well plates. After 24 h, the cells were co-transfected with the luciferase reporter plasmid and the snail promoter or PGL4.48. Firefly and Renilla luciferase activities were measured 24 h post-transfection using the DualGlo Luciferase Assay System (Promega, USA). Firefly luciferase activity was normalized to that of Renilla luciferase. All experiments were performed three times.

### IHC and immunofluorescence (IF) analysis

IHC analysis was performed as described previously [24, 25]. The microscopic examination of each point of the tissue microarray was performed at the same incident light intensity and compensation intensity. The total score for each case was calculated as the product of the staining intensity score and the stained positive cells score. The rule for the staining intensity score: 0 points (Negative); 1 point (Light brown); 2 points (Brown); 3 points (Dark brown). The rule for the stained positive cells score: 0 points (0%); 1 point (10–25%); 2 points (26–50%); 3 points (51–75%); 4 points (76–100%). High expression was recorded if the total score was more than or equal to 6 points, otherwise the result was regarded as low expression. The scoring of the tissue chip was independently conducted by two pathologists who were blinded to the patients’ clinical case data. IF staining was performed as previously reported [26]. DEPTOR, E-cadherin and occluding antibody were all diluted to 1:100. Nuclei were labeled with 4′,6-diamidino-2-phenylindole.

### Animal studies

Xenograft tumorigenicity assays were performed as described previously [27]. All in vivo studies were performed in compliance with the National Institutes of Health guidelines (NIH publication 86–23 revised 1985) and approved by the Committee on the Ethics of Animal Experiments of Tongji Medical College, Huazhong University of Science and Technology. For the lung metastasis assay, mice were injected intravenously with 1 × 10^6^ of the indicated tumor cells suspended in serum-free medium via the lateral tail vein. All mice were sacrificed after 6–8 weeks and the lung tissues were removed and fixed in 4% phosphate-buffered neutral formalin for 72 h. Then, the metastatic lungs were longitudinal sectioned every 0.5 mm, so that approximately 20 slices could be cut for each lung. Metastasis foci were quantified by H&E staining.

### Statistical analysis

The data were analyzed using GraphPad Prism 5.0. All experiments were performed independently at least three times, and the results were presented as the means ± SEM. Categorical data were analyzed using the χ2 test, and quantitative data were compared using the two-tailed Student’s *t*-test or analysis of variance (ANOVA) with the Tukey-Kramer multiple comparisons test. A two-tailed *P-*value < 0.05 was considered to indicate statistical significance for all tests (Additional file 3).

## Results

### DEPTOR was highly expressed in HCC

**Fig. 1:**
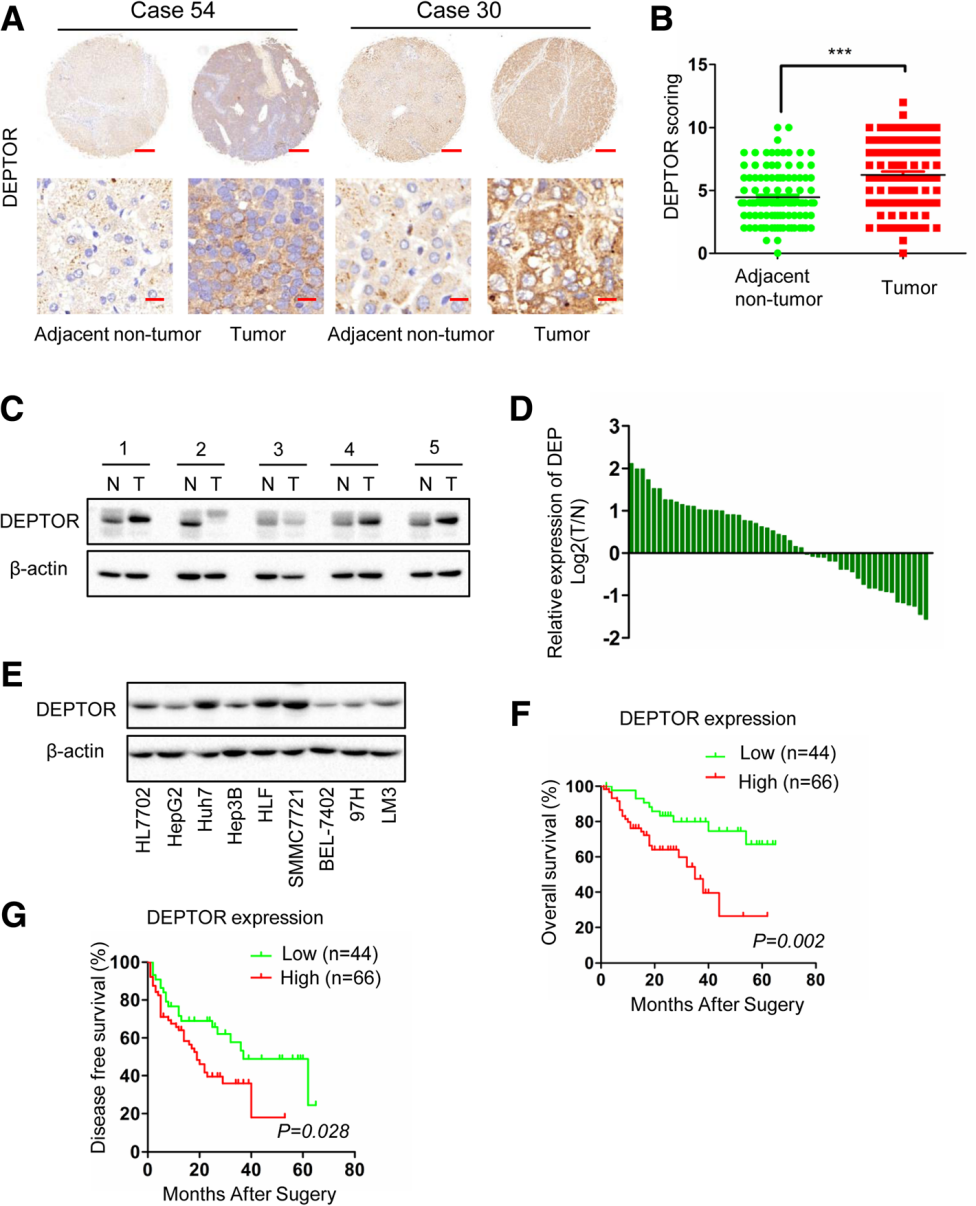
DEPTOR is overexpressed in HCC, and high expression of DEPTOR is associated with a poor prognosis in HCC patients. **a** IHC analysis of DEPTOR expression in 110 paired HCC tissues and adjacent non-tumor tissues. Representative images of DEPTOR expression are shown in the left panels. Scale bar, 300 μm (upper panel) or 20 μm (lower panel). **b** Statistical analysis of DEPTOR expression in HCC tissues and adjacent non-tumor tissues. **c** Western blot analysis of relative DEPTOR expression in 53 HCC tissues (T) and adjacent non-tumor tissues (N). **d** DEPTOR bands of HCC tissues were quantified and shown in the bar chart after being normalized to the respective adjacent non-tumor tissues. **e** Western blot analysis of DEPTOR expression in a normal liver cell line and eight HCC cell lines. **f** Kaplan-Meier analysis of the correlation between DEPTOR expression and overall survival (OS) of HCC patients. **g** Kaplan-Meier analysis of the correlation between DEPTOR expression and disease-free survival (**g**) of HCC patients

To explore the possible role of DEPTOR in HCC, we first detected its expression in a tissue microarray of 110 pairs of HCC and adjacent non-tumor tissues by IHC staining. The IHC assays showed that DEPTOR was overexpressed in 60% (66/110) of HCC tissues and presented low expression in 40% (44/110) of HCC tissues compared with adjacent non-tumor tissues. The results of IHC scoring showed that the average expression level of DEPTOR was significantly higher in HCCs than in adjacent non-tumor tissues (Fig.1a and b). We then performed western blot analysis to measure the expression of DEPTOR in 53 pairs of HCC tissues vs. adjacent non-tumor tissues (Fig. 1c and Additional file 4: Figure S1A). Consistent with the former results, 58.5% (31/53) of HCC tissues showed higher expression of DEPTOR than adjacent non-tumor tissues (Fig. 1c, d and Additional file 4: Figure S1A). Next, western blot was performed to measure the expression of DEPTOR in normal liver cell lines and HCC cell lines. DEPTOR showed high expression in Huh7, HLF and SMMC-7721 cells and low expression in the normal cell line HL7702 and other HCC cell lines (Fig. 1e). Therefore, the degree of DEPTOR expression was highly variable among different HCC cell lines.

### High expression of DEPTOR was associated with aggressive tumor behavior and poor prognosis

The IHC results showed that the average expression of DEPTOR was significantly higher in HCCs. To assess the clinical significance of DEPTOR expression, eleven established factors of HCC malignancy were analyzed. We found that high DEPTOR levels were significantly associated with serum AFP levels (*p* = 0.035), tumor size (*p* = 0.048), vascular invasion (*p* = 0.020), TMN stage (*p* = 0.033) and BCLC stage (*p* = 0.048) of HCC (Additional file 1: Table S1). Since vascular invasion frequently indicates the ability of tumor metastasis, the results suggest that DEPTOR may be involved in HCC metastasis. HCC patients with high expression of DEPTOR had a shorter overall survival (OS, *p* = 0.002) and disease-free survival (DFS, *p* = 0.028) than those with low expression of DEPTOR (Fig. 1f and g). These results therefore demonstrated that DEPTOR expression was closely correlated with aggressive tumor behavior and poor survival in HCC patients.

### Knockdown of DEPTOR inhibited the proliferation of HCC cells in vitro and in vivo

**Fig. 2:**
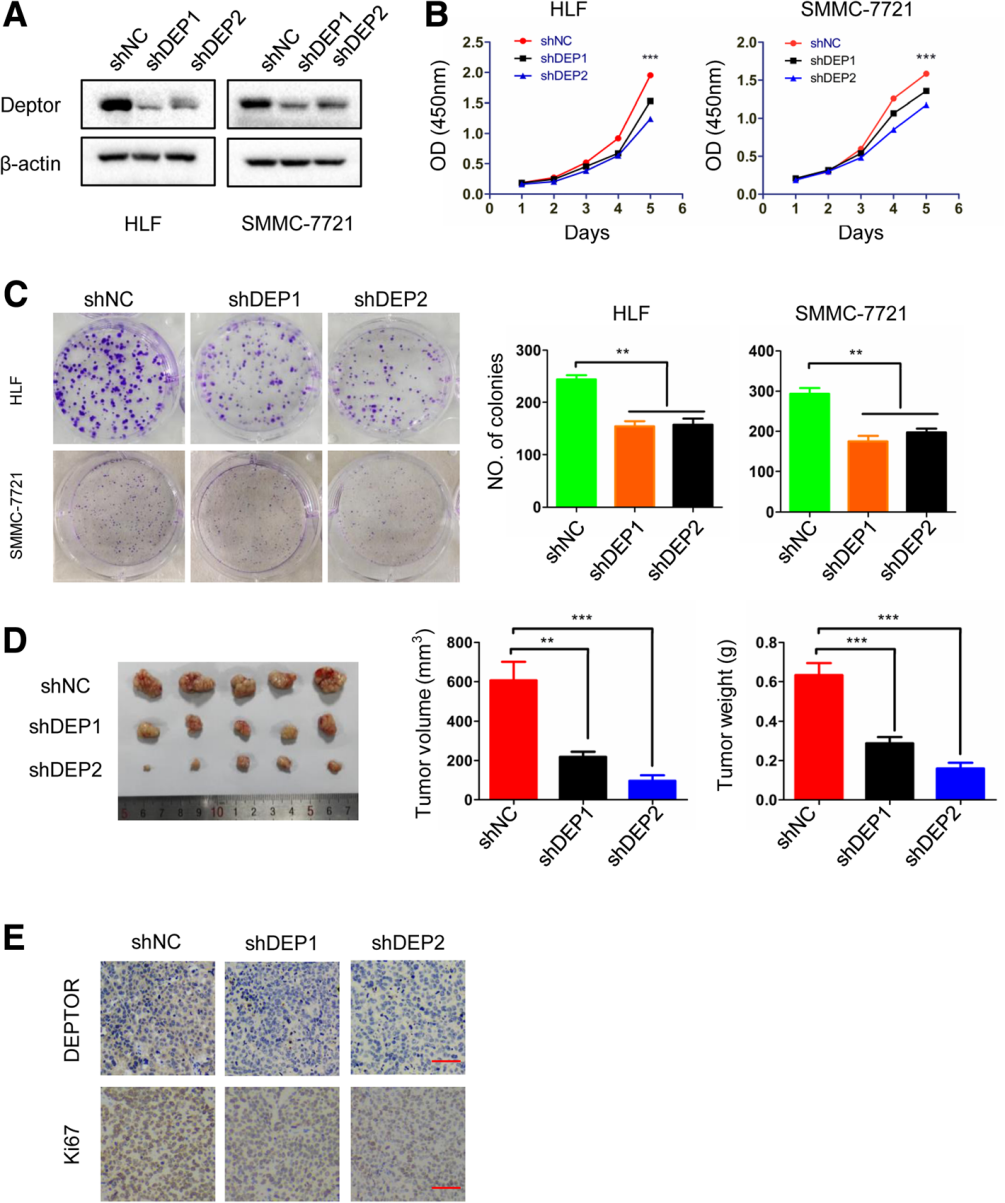
DEPTOR depletion inhibits the proliferation of HCC cells in vitro and in vivo. **a** HLF and SMMC-7721 cells were transfected with shRNAs against DEPTOR or shNC control lentivirus. Western blot analysis was used to determine the knockdown efficiency. **b** The indicated HCC cell lines were subjected to the CCK8 assay. **c** The indicated HCC cell lines were subjected to colony formation assays. Representative images are shown (left panel) and statistical comparisons of the indicated groups were performed (right panel). **d** Subcutaneous tumors composed of HLF-shDEP1/2 cells and the control cells are shown in the left panels. Tumor volume is shown in the middle panels. Tumor weight is shown in the right panels. **e** IHC staining for DEPTOR and Ki-67 expression in xenograft tumors of different groups. Scale bar, 50 μm. The data represent the means ± SEM from three independent experiments. *P < 0.05, **P < 0.01, ***P < 0.001

We next investigated the functions of DEPTOR in HCC cells using in vivo and in vitro assays. We measured the endogenous expression of DEPTOR in liver cells (HL7702) and 8 HCC cell lines. We chose the SMMC-7721 and HLF cell lines with high metastatic and invasive capabilities for knockdown experiments. Three short hairpin RNAs (shRNA#1, shRNA#2 and shRNA#3) were designed to silence the expression of DEPTOR in SMMC-7721 and HLF cells, resulting in the cell lines SMMC-7721-shNC, SMMC-shDEP1–3, HLF-shNC and HLF-shDEP1–3, respectively. Western blot analysis was used to confirm the knockdown efficiency. The shRNA#1 and shRNA#2 had significant knockdown effects and were chosen for further study (Fig. 2a). To explore the effect of DEPTOR on cell proliferation in vitro, we performed the cell counting kit 8 (CCK8) assay to evaluate the proliferation of HCC cells following DEPTOR silencing. We found that knockdown of DEPTOR significantly reduced the proliferation rate of SMMC-7721 and HLF cells compared to the non-target shNC control cells (Fig. 2b). The inhibitory effect of DEPTOR silencing on cell growth was further confirmed using a colony formation assay. As shown in Fig. 2c, downregulation of DEPTOR by shRNAs reduced the number and size of colonies compared to the shNC control cells. We then performed animal experiments to determine the effect of DEPTOR downregulation on tumor growth in vivo. In a subcutaneous xenograft nude mouse model, the animals injected with shRNA-transfected HLF cells (HLF shDEP1 and HLF shDEP2) were found to have tumors of much smaller size and lower weight than those injected with shNC-transfected HLF cells (Fig. 2d). Moreover, IHC staining of the xenograft tumors showed that the expression of DEPTOR and the proliferation marker Ki-67 was significantly decreased in the shDEP1 and shDEP2 group compared to the shNC group (Fig. 2e). Taken together, these results fully demonstrate that knockdown of DEPTOR significantly inhibits the proliferation of HCC cells.

DEPTOR presents low expression both in less aggressive HCC cell lines (e.g. HL7702, HepG2, Hep3B) and highly aggressive ones (e.g. BEL-7402, MHCC-97H and HCC-LM3). Therefore, both high aggressive HepG2 and less aggressive BEL-7402 cells were stably transfected with a DEPTOR expression construct and empty control, resulting in the DEP and VEC stable expression lines, respectively. Western blot analysis was performed to validate the effect of overexpression (Additional file 4: Figure S2A). Consistent with a previous report [19], the CCK8 and colony formation assays showed that overexpression of DEPTOR had little effect on cell proliferation or colony formation (Additional file 4: Figure S2B).

### DEPTOR promotes the mobility and metastasis of HCC cells in vitro and in vivo

**Fig. 3:**
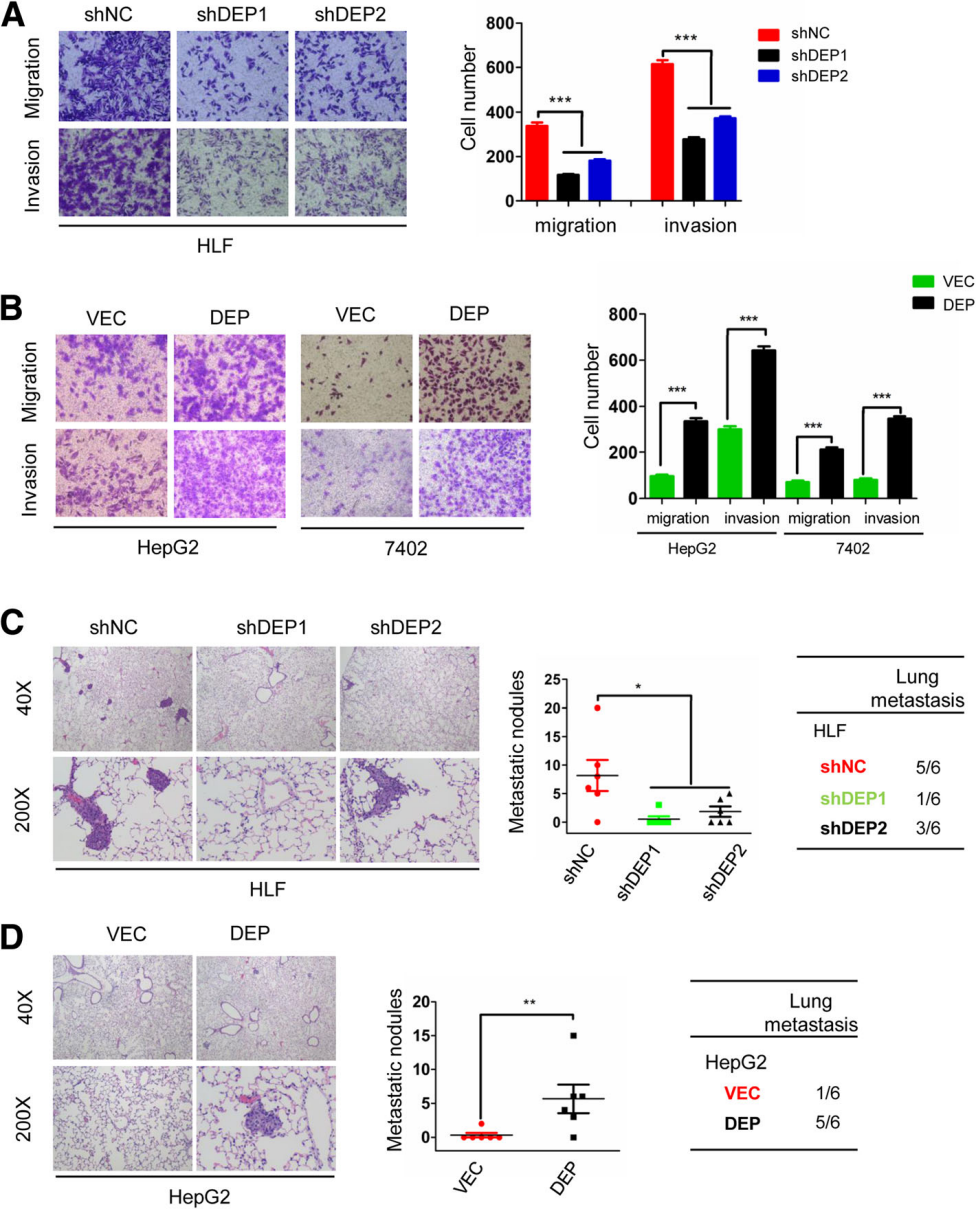
DEPTOR promoted the invasion and metastasis of HCC cells in vitro and in vivo. **a** Knockdown of DEPTOR expression reduced the migration and invasion of HLF cells in the transwell assay. **b** DEPTOR overexpression enhanced migration and invasion of HepG2 and 7402 cells in the transwell assay. Representative images of lung metastases derived from HLF-shDEP1/2 and the control cells (**c**) or HepG2-DEP and the corresponding control cells (**d**) are shown in the left panels, the proportion of metastatic nodules in the lungs was calculated as shown in the middle panels. The rate of lung metastasis is shown in the right panels. The data represent the means ± SEM from three independent experiments. *P < 0.05, **P < 0.01, ***P < 0.001

We also performed transwell assays to test the effect of DEPTOR knockdown or overexpression on HCC cell mobility. The results showed that DEPTOR downregulation significantly impaired the migration and invasion capacity of HLF cells compared to its shNC control (Fig. 3a), while overexpression of DEPTOR either in BEL-7402 cells or HepG2 cells enhanced the migration and invasion ability compared to its VEC control (Fig. 3b).

We further examined the role of DEPTOR in HCC metastasis by establishing an ectopic tumor metastasis model in nude mice. The nude mice were injected with HLF shDEP1, HLF shDEP2 and HLF shNC cells, as wells as HepG2-VEC and HepG2-DEP cells into the tail vein, respectively. The animals were sacrificed after 6 weeks, the lungs were removed, and consecutive sections were taken from every lung tissue block and stained with H&E. The number of metastatic foci derived from HLF shDEP1 and shDEP2 cells and the corresponding lung metastasis rate were significantly decreased compared to those of HLF shNC cells (Fig. 3c). Conversely, a significantly higher number of metastatic foci and higher lung metastasis rate were found in the HepG2-DEP injection group compared to the HepG2-VEC group (Fig. 3d). These data confirm that DEPTOR promotes the mobility and metastasis of HCC cells in vitro and in vivo.

### DEPTOR induces the EMT and mobility in HCC cells by snail induction

**Fig. 4:**
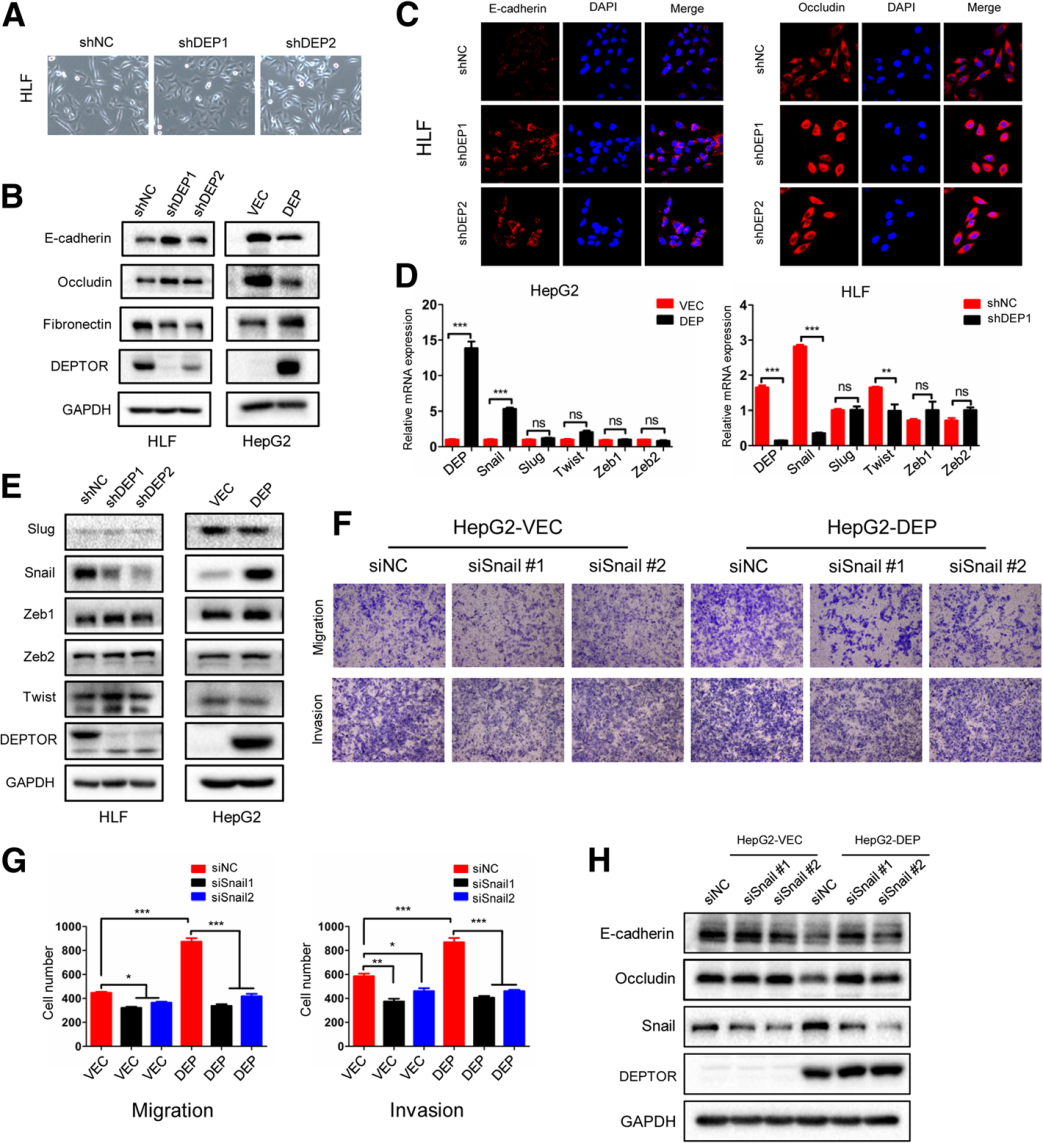
DEPTOR promoted the EMT, migration and invasion of HCC cells via snail induction. **a** Representative phase contrast images of HLF-shDEP1/2 cells and the corresponding control cells. **b** The expression of EMT markers mediated by DEPTOR knockdown or overexpression was detected by western blotting. **c** IF staining for E-cadherin and occluding in HLF-shDEP1/2 cells and the corresponding control cells. Scale bar: 30 μm. **d** The mRNA expression of EMT-TFs was determined in the indicated cells. **e** The influence of DEPTOR knockdown or overexpression on the expression of EMT-TFs as detected by western blot. **f** Two siRNAs targeting snail effectively decreased snail expression in HepG2-DEP cells and the control cells. The transwell assay was used to detect the capacity of migration and invasion in the indicated cells following snail knockdown. **g** Statistical comparison of the indicated groups was performed. **h** Knockdown of snail expression reversed the DEPTOR-induced EMT. The data represent means ± SEM from three independent experiments. *P < 0.05, **P < 0.01, ***P < 0.001

The epithelial-to-mesenchymal transition (EMT) is a key step in tumor metastasis [6]. Obvious morphological changes were observed in the tested cell lines following DEPTOR knockdown and overexpression. HLF shNC cells exhibited spindle-like mesenchymal cell morphology, while HLF shDEP1 and shDEP2 cells mostly changed into an epithelial cell morphology (Fig. 4a). Conversely, HepG2 cells changed from an epithelial morphology to a mesenchymal morphology after stable transfection with the DEPTOR expression construct (Additional file 4: Figure S3A). Consequently, we proposed that DEPTOR promotes the EMT in HCC cells. To further validate this conjecture, western blot and immunofluorescence (IF) analysis were performed to detect the expression of EMT-related markers. We found that E-cadherin and occludin were upregulated, while fibronectin was downregulated by DEPTOR knockdown in HLF cells (Fig. 4b). Moreover, the opposite expression pattern was observed in HepG2 cells with DEPTOR overexpression (Fig. 4b). The IF results confirmed that DEPTOR was efficiently knocked down (Additional file 4: Figure S3B) and showed that the fluorescence intensity of E-cadherin and occludin were markedly increased in shDEP1 and shDEP2-transfected HLF cells compared to shNC-transfected HLF cells (Fig. 4c). Correspondingly, an obvious morphological switch from the mesenchymal to the epithelial phenotype was observed after DEPTOR knockdown in HLF cells (Fig. 4c). To investigate the correlation between DEPTOR and E-cadherin in clinical samples, we analyzed the expression of DEPTOR and E-cadherin by IHC on a tissue microarray containing 60 pairs of HCC samples. The results showed that expression of DEPTOR was negatively correlated with E-cadherin in the clinical samples (*r* = − 0.286, *P* = 0.027, Pearson correlation, respectively) (Additional file 4: Figure S3E). Overall, these data demonstrate that DEPTOR promotes the EMT in HCC cells.

EMT-related transcription factors (EMT-TFs) are important triggers of the EMT, with Snail, slug, ZEB1, ZEB2 and twist as important members of this group [6]. To investigate whether DEPTOR-induced EMT was associated with these five EMT-TFs, Real time PCR and western blot analysis were performed to detect their expression. The results showed that only snail was significantly increased in HepG2-DEP cells compared to HepG2-VEC cells (Fig. 4d). A similar result was observed in HLF cells after DEPTOR knockdown (Fig. 4d). Consistently, snail expression was also changed at the protein level in response to either DEPTOR knockdown or DEPTOR overexpression in HCC cells, while that of the other tested TFs was not changed (Fig. 4e). To further investigate whether the DEPTOR-induced EMT and increase of cell mobility were related to snail induction, we used two siRNAs to block the expression of snail in HepG2-DEP cells. After snail silence in HepG2-DEP cells, the capacity of promoting cell migration and invasion by DEPTOR was significantly impaired (Fig. 4f and g). Similarly, DEPTOR-inducing EMT was reversed by snail knockdown (Fig. 4h), while overexpression of snail significantly promoted the migration, invasion and EMT in HLF-shDEP1 cells (Additional file 4: Figure S3C and S3D). These data confirm that DEPTOR promoted HCC invasion and EMT by snail induction.

### DEPTOR promotes autocrine TGF-β1 signaling by mTOR inhibition

**Fig. 5:**
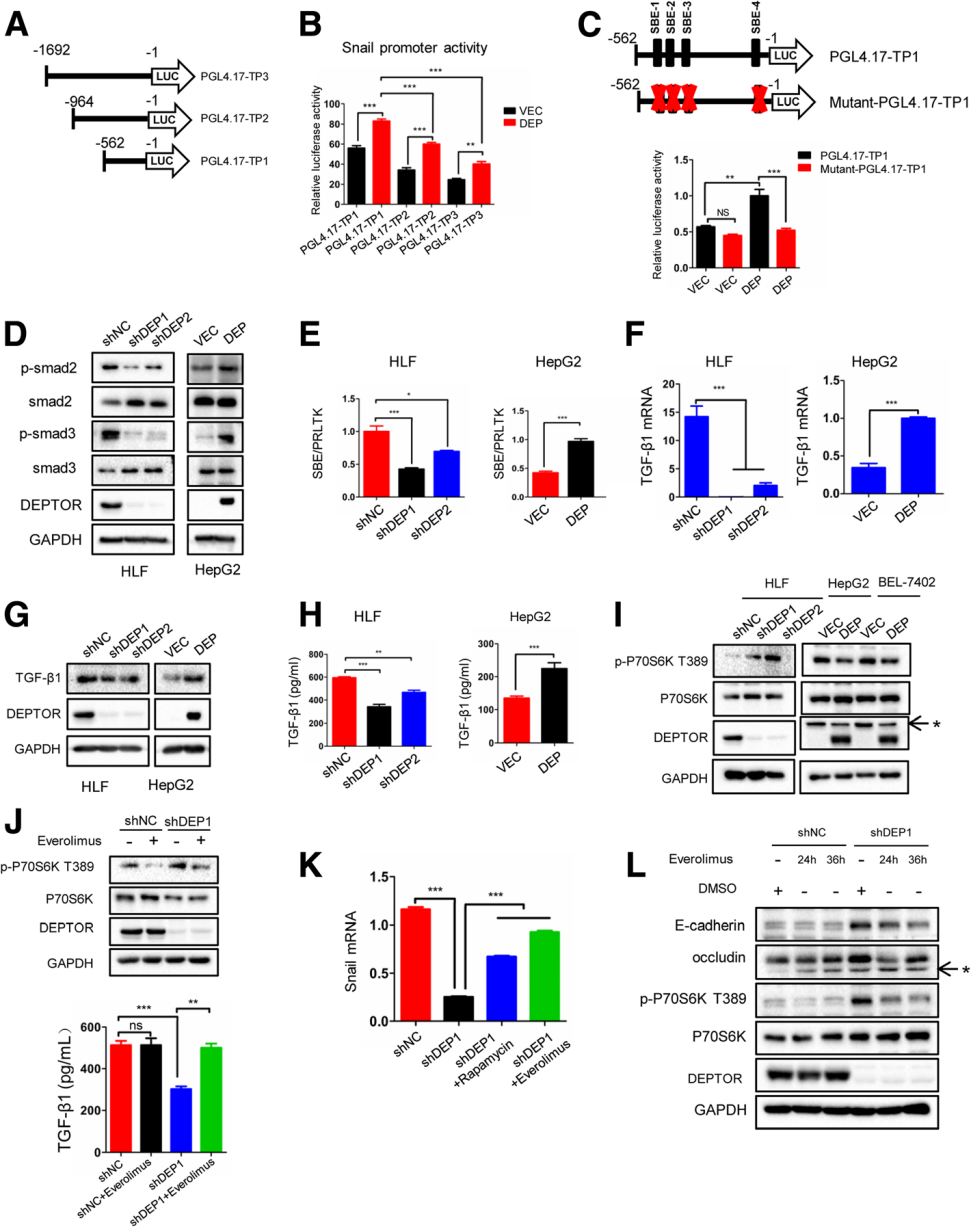
DEPTOR possibly promotes autocrine TGF-β1 signaling via mTOR inhibition. **a** A series of 5′-truncated snail promoter luciferase constructs were prepared and verified by sequencing. **b** The results of luciferase reporter assays using HepG2-DEP cells and control cells. HepG2-DEP or HepG2-VEC cells were transiently transfected with the indicated constructs and their luciferase activities were determined after 24 h. **c** The cells were transiently transfected with the constructs including the wild-type or mutated Smad-binding sites. The relative luciferase activities of the constructs are shown. **d** Key members of the TGF-β signaling pathway were detected by western blotting. **e** The luciferase activity of constructs with the indicated Smad-binding elements was detected in the indicated cells. **f** The mRNA expression of TGF-β1 was detected in the indicated cells. **g** TGF-β1 protein was detected in the indicated cells. **h** The secretion of TGF-β1 was detected by ELISE in the indicated cells. **i** The expression of p-P70S6K (T389) and P70S6K was detected by western blotting in the indicated cells. **j** The indicated cells with everolimus or left untreated for 24 h. The expression of p-P70S6K (T389) and P70S6K as detected by western blotting and the secretion of TGF-β1 was detected by ELISA. **k** HLF-shDEP1 cells were treated with rapamycin and everolimus for 24 h and snail mRNA was detected by RT-PCR. **l** The indicated cells were treated with everolimus or left untreated for the indicated periods, after which E-cadherin, occludin, p-P70S6K (T389), P70S6K and DEPTOR were detected by western blotting. The data represent the means ± SEM from three independent experiments. *P < 0.05, **P < 0.01, ***P < 0.001. * was indicated as non-specific band

The observation that DEPTOR induces EMT by upregulating snail at the mRNA and protein levels suggested that snail was upregulated at the transcriptional level. To search for possible transcription factors involved in snail upregulation, we selected a ~ 1.6-kb 5′-region of the snail promoter to generate a series of luciferase reporter constructs with truncations of different lengths (Fig. 5a) and measured their basal luciferase activity in HepG2-VEC and HepG2-DEP cells. As shown in Fig. 5b, the luciferase activity of both full-length and truncated snail promoter constructs was increased in HLF-DEP cells compared to the HLF-VEC controls. Furthermore, the luciferase activity of PGL4.17-TP1 was higher than that of PGL4.17-TP2 and TP3 either in HepG2-VEC or HepG2-DEP cells, suggesting that an inhibitory element exists at positions − 562 to − 1692 in the snail promoter and that DEPTOR promotes snail transcription mainly via elements at positions − 1 to − 562. We then used jasper (http://jaspar.genereg.net/) to predict possible transcription-factor binding sites in this region. Interestingly, we found four Smad-binding elements (SBE) upstream of the transcriptional start site (TSS) (Additional file 4: Figure S4). Moreover, snail was found to be upregulated at the transcriptional level through the TGF-β/Smad pathway [16]. Therefore, we speculated that the increased expression of snail was due to the activation of the TGF-β1/Smad pathway. To validate this hypothesis, we mutated all the SBEs (Fig. 5c and Additional file 4: Figure S4) and the corresponding luciferase reporter assay showed that the mutant-PGL4.17 TP1 exhibited a two-fold reduction of luciferase activity compared to its wild-type parent construct (Fig. 5c). These data suggest that DEPTOR-induced snail upregulation is associated with Smad pathway activation.

We then performed western blot analysis to investigate the phosphorylation state of Smad3 and Smad2, which are markers of the activation of the canonical Smad pathway [17]. The phosphorylation of Smad3 and Smad2 was decreased or increased in response to DEPTOR knockdown or overexpression, respectively (Fig. 5d). This result was further confirmed by the luciferase reporter assay. As shown in Fig. 5e, the activity of the Smad-binding elements (SBE-luc) was significantly downregulated in HLF shDEP1 and shDEP2 cells compared to HLF shNC cells (shNC vs. shDEP1:1.00 ± 0.16 vs. 0.43 ± 0.03, *p* < 0.001; shNC vs. shDEP2: 1.00 ± 0.06 vs. 0.70 ± 0.02, *p* < 0.05). Conversely, SBE-luc activity was higher in HepG2-DEP cells than in HepG2-VEC cells (HepG2-VEC vs. HepG2-Dep: 0.44 ± 0.03 vs. 1.00 ± 0.06, *p* < 0.001). Taken together, these data confirm that DEPTOR promotes the activation of the Smad pathway in HCC cells.

Smad activation is mediated by receptor-ligand binding, and TGF-β1 binds with the TβR-II type II receptor and subsequently propagates canonical TGF-β signaling through phosphorylation of the receptor-associated Smads [12]. To investigate whether DEPTOR-induced Smad activation was a result of TGF-β1 production, RT-PCR and western blot analyses were performed. The results showed that the mRNA and protein levels of TGF-β1 were significantly downregulated in HLF-shDEP1 and HLF-shDEP2 cells compared to the HLF-shNC control, while they were increased in HepG2-DEP cells compared to HepG2-VEC cells (Fig. 5f and g). We further performed ELISA to measure the secretion of TGF-β1. We found that TGF-β1 secretion into the extracellular space was markedly reduced in HLF shDEP1 and HLF shDEP2 cells compared to HLF shNC cells (HLF shNC vs. HLF shDEP1: 594.2 ± 13.7 vs. 341.2 ± 40.7, *p* < 0.001; HLF shNC vs. HLF shDEP1: 594.2 ± 13.7 vs. 466.9 ± 23.0, *p* < 0.01). By contrast, the secretion of TGF-β1 was much higher in HepG2-DEP cells than in HepG2-VEC cells (HepG2-VEC vs. HepG2-DEP: 134.9 ± 8.0 vs. 224.6 ± 26.3, *p* < 0.001) (Fig. 5h). These results all point in the direction that DEPTOR mediates Smad activation by promoting the secretion of TGF-β1.

DEPTOR was initially identified as a factor that inhibits mTORC1 and mTORC2 by direct binding to mTOR [18]. Consistently with previous studies, we found that mTOR was activated by DEPTOR knockdown or deactivated upon DEPTOR overexpression by detecting the phosphorylation state of its downstream target P70S6K (Fig. 5i). To further investigate whether DEPTOR-induced autocrine TGF-β1 signaling was associated with its function of mTOR inhibition, we treated HLF shDEP1 cells which possess high mTOR activity with the mTORC1 inhibitor everolimus for 24 h. As shown in Fig. 5j, everolimus efficiently eliminated mTOR activity and inhibited the secretion of TGF-β1 in HLF shDEP1. This suggests that mTOR activity is necessary for TGF-β1 production and secretion. As demonstrated in the aforementioned experiments, the mRNA expression of snail was reduced in HLF shDEP1 cells compared to HLF shNC cells. Rapamycin (sirolimus), a classic inhibitor of mTORC1, and its 40-O-(2-hydroxyethyl) derivative everolimus can reverse snail mRNA expression in HLF shDEP1 cells (Fig. 5k). Correspondingly, everolimus significantly reduced the phosphorylation of p70s6k and the expression of E-cadherin and occludin in HLF shDEP1 cells (Fig. 5l). These results suggest that DEPTOR possibly promotes TGF-β1 secretion via a feedback mechanism related to the inhibition of mTOR activity.

### Blocking the TGF-β1-Smad3/Smad4 pathway inhibits the DEPTOR-induced EMT and invasion

**Fig. 6:**
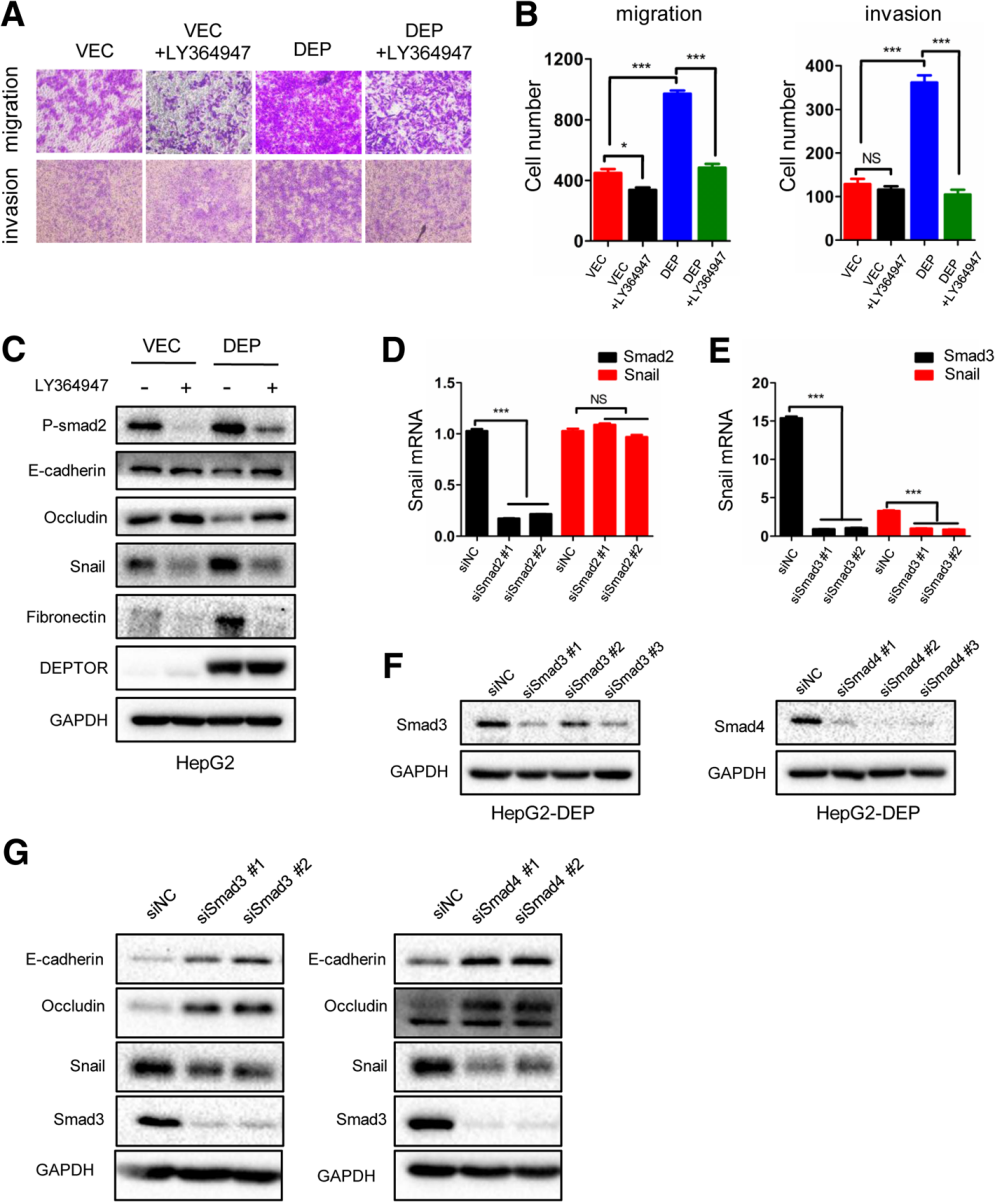
Blocking the TGF-β1-Smad3/Smad4 signaling pathway inhibited the DEPTOR-induced EMT and invasion. **a**, **b** The indicated cells were treated with LY364947 or left untreated for 24 h and then subjected to the transwell assay. Statistical comparisons of the indicated groups were performed (right panel). **c** The indicated cells were treated with LY364947 or left untreated for 24 h and then subjected to western blotting to detect EMT markers. **d** HepG2-DEP cells were transfected with siRNA against smad2 for 48 h and then subjected to RT-PCR for detecting the expression of snail and smad2 mRNA. **e** HepG2-DEP cells were transfected with siRNA against smad3 for 48 h and then subjected to RT-PCR for detecting the expression of snail and smad3 mRNA. **f** Western blotting was performed to detect the expression of EMT markers after smad3 or smad4 knockdown. The data represent the means ± SEM from three independent experiments. *P < 0.05, **P < 0.01, ***P < 0.001

After demonstrating that DEPTOR upregulated TGF-β1 expression at the mRNA and protein levels, we further tested the role of TGF-β1 in the DEPTOR-induced cell mobility and EMT by first measuring the invasiveness of HepG2-VEC and HepG2-DEP cells with or without the TβR1 inhibitor, LY364947. The transwell assay showed that LY364947 treatment significantly decreased the migration and invasion capacity of HepG2-DEP cells with high expression of DEPTOR and TGF-β1, but had none or a weak effect on HepG2-VEC cells (Fig. 6a and b). Western blot analysis revealed that LY364947 treatment increased the expression of epithelial markers (E-cadherin and occludin) and decreased the expression of mesenchymal markers (snail and fibronectin) in HepG2-DEP cells, but these EMT markers were not markedly changed in response to LY364947 treatment in HepG2-VEC cells (Fig. 6c). These results further confirm that DEPTOR induces cell invasion and EMT via TGF-β1 secretion.

TGFβ-type cytokines primarily bind to the type II receptor (TβRII) and promote recruitment of the type I receptors (TβRI or ALK5) [28]. The activated TβRI then phosphorylates the intracellular effector Smads (R-Smad for receptor-activated Smads, e.g. Smad2 and Smad3), resulting in their activation and association with co-Smad (Smad4). The resulting trimeric complexes then translocate into the nucleus and regulate gene transcription [28]. However, abundant evidence suggests that R-Smads play different roles in cancer progression by controlling distinct transcriptional programs [13]. To investigate the role of R-Smads in DEPTOR-induced EMT, we used siRNA to silence the expression of Smad2 and Smad3. RT-PCR analysis was performed to confirm the knockdown efficiency of Smad2 and Smad3 and detect the mRNA expression of snail. The results showed that only Smad3 silencing decreased snail mRNA expression, while Smad2 silencing had no effect (Fig. 6d and e). Western blot analysis also supported this conclusion. We found that Smad3 knockdown decreased the protein expression of snail and increased the expression of E-cadherin and occludin (Fig. 6f). Interestingly, similar results were observed when Smad4 was knocked down (Fig. 6f). This suggests that the Smad3/Smad4 complex, and not the Smad2/Smad3/Smad4 complex, is involved in DEPTOR-induced snail upregulation.

## Discussion

DEPTOR contains two DEP domains and one PDZ domain, whereby the latter interacts with mTOR to inhibit its activity [18]. In this study, we demonstrated that DEPTOR was frequently overexpressed in HCC tissues. Moreover, high expression of DEPTOR was associated with high serum AFP levels, increased tumor size, vascular invasion and more advanced TMN and BCLC stage. In addition, patients with high expression of DEPTOR had worse prognosis than those with low expression. Both in vivo and in vitro functional experiments demonstrated that DEPTOR promotes the EMT and metastasis of HCC cells by activating the TGF-β1-smad3/smad4-snail pathway. DEPTOR overexpression possibly relieved the mTORC1-mediated inhibition of TGF-β signaling by mTOR inhibition. Therefore, targeting DEPTOR may be an ideal treatment strategy for inhibiting HCC metastasis and prolonging the survival of patients with HCC.

Hyperactivation of mTORC1 due to mutation of its upstream oncogenic pathways, including the PI3K/AKT and RAS/RAF/MEK/ERK pathways, is present in numerous human cancers and mainly involved in tumor growth and survival [29]. DEPTOR is a direct endogenous inhibitor of mTORC1 and mTORC2 that is overexpressed in various cancers, such as colorectal cancer [23], osteosarcoma [20], myeloma [18], cervical squamous cell carcinoma [21] and HCC [19]. DEPTOR significantly promotes the growth of tumor cells in vivo and in vitro by inhibiting mTOR, which releases the negative feedback on the insulin PI3K/AKT pathway and therefore promotes cell survival and prevents apoptosis [18–21]. Paradoxically, DEPTOR acts as a tumor suppressor in lung adenocarcinoma and multiple myeloma [30, 31]. DEPTOR presented low expression in tumor samples compared to adjacent non-tumor samples and was found to inhibit tumor cell proliferation in vitro and in vivo by promoting EGFR degradation in lung adenocarcinoma [30]. In multiple myeloma, DEPTOR maintains differentiation, and its high expression predicts a better prognosis [31]. In our study, the functional experiments revealed that knockdown of DEPTOR in HLF and SMMC-7721 cells significantly suppressed their proliferation in vivo and in vitro. Consistent with previous reports on HCC [19], overexpression of DEPTOR had no effect on cell growth in various HCC cell lines. The reason for this phenomenon is still not clear. Possible explanations for this difference include, but are not limited to, different tumor origins and/or molecular effectors leading to cell growth in diverse HCC cell lines.

To date, only few studies focused on the role of DEPTOR in tumor metastasis [22, 32]. For example, DEPTOR was found to enhance the metastasis of triple-negative breast cancer in vivo by upregulating the expression of survivin [22]. However, the role of DEPTOR in HCC metastasis has not been elucidated to date. Our study showed that DEPTOR overexpression significantly promoted the invasion, metastasis and EMT of HCC cells. During the initial stages of metastasis, the tumor cells undergo the EMT, which contributes to their migration and invasion [6]. Then, the tumor cells are able to disassociate from the primary tumor, intravasate into blood vessels, and finally metastasize to distant organs [33]. Classical EMT results in the transition of epithelial (E) cells to cells with a mesenchymal (M) phenotype, defined by prototypical markers such as E-cadherin and vimentin [6, 33]. The link between DEPTOR and the EMT was reported recently. DEPTOR inhibits the EMT process by inhibiting the AKT/GSK3β/snail pathway in lung carcinoma cells [32]. Interestingly, DEPTOR can inhibit the activation of the AKT pathway in this context, which contradicts many earlier reports [32], and a recent report found that DEPTOR is essential for tumor metastasis in triple-negative breast cancer [22]. However, DEPTOR partially suppresses the EMT in breast cancer cells [22].

The EMT is triggered by so-called EMT-activating transcriptional factors (EMT-TFs) such as snail, slug, Zeb1, Zeb2 and twist [33]. In the present study, we found that DEPTOR overexpression induced snail mRNA and protein expression, while that of the other four tested EMT-TFs was not changed. As an important transcription factor of the EMT, snail directly binds to the E-cadherin promoter and inhibits its expression [11]. We consequently used siRNA to silence the expression of snail, and found that this intervention reversed the DEPTOR-induced EMT and invasion in HepG2 cells. Thus, we concluded that the DEPTOR-induced EMT and invasion are mediated by snail induction.

Multiple signaling pathways, including TGF-β [34, 35], Wnt [36], and Notch pathways [37, 38], reactive oxygen species [39, 40], and hypoxic stress [41, 42], have been found to induce snail expression. Among them, TGF-β signaling is one of the most prominent EMT-inducing cytokine pathways that upregulates snail expression [43]. Smad3/4 complexes bind to the snail promoter close to the TSS in response to TGF-β1 treatment [35]. In the study, we found that DEPTOR promoted the mRNA and protein expression of TGF-β1, thus leading to its increased secretion. In addition, LY364947, a TβR1 inhibitor, had a smaller or no effect on the invasion and EMT of HepG2-VEC cells because of the low expression of endogenous TGF-β1 [25]. However, LY364947 significantly impaired the migration and invasion capacity of HepG2-DEP cells and reversed their EMT. These results further confirmed that DEPTOR induced the invasion and EMT of HCC cells via TGF-β1 signaling.

Multiple studies report that there may be a negative feedback between mTOR and TGF-β signaling [44, 45]. The silencing of mTOR in the normal epidermis inhibits cell differentiation, and impairs cell-cell adhesion by promoting TGF-β signaling via increased TGF-β receptor (ALK5) expression [45]. Furthermore, silencing of mTOR signaling by a knockdown of its components increases TGF-β signaling in hepatic progenitor cells [44]. We found that both rapamycin and everolimus promoted the EMT in HLF shDEP cells with high mTOR activity. We considered that they have the same effect because of their function of mTOR inhibition. Despite this, we still lack direct evidence to prove this conclusion. Pharmacological disruption of the DEPTOR-mTOR interaction [46] needs to be further investigated. The study suggests that single mTOR inhibitors may not be an ideal choice for treating cancer. Although mTOR inhibitors can slow tumor growth, they have the potential to promote metastasis by activating TGF-β signaling. However, additional in vivo experiments in different metastasis models are needed to further clarify the risk of metastasis due to mTOR inhibition by rapamycin or everolimus. Clinically, a combination of mTOR inhibitors and TGF-β inhibitors may be a better therapeutic strategy for targeting HCC.

## Conclusions

DEPTOR promotes the EMT and metastasis HCC cells by activating the TGF-β1-smad3/smad4-snail pathway via mTOR inhibition. Consequently, targeting DEPTOR may be an ideal treatment strategy for inhibiting HCC growth and metastasis.

## Additional files

Additional file 1: Association of DEPTOR expression with Clinicopathologic Features in 110 Primary HCCs. (TIF 225 kb)

Additional file 2: The target sequences of shRNA and siRNA are listed in the table. (TIF 156 kb)

Additional file 3: The indicated primers used in the study were showed in the table. (TIF 241 kb)

Additional file 4: **Figure S1.** Western blotting analysis of relative DEPTOR expression in 53 HCC tissues (T) and its adjacent non-tumor tissues (N). **Figure S2.** (A) Western blotting was used to detect the overexpression efficiency of 7402 and HepG2 cells. (B) Proliferation of 7402-DEP, HepG2-DEP cells and control cells were examined by CCK8 assay. (C) Proliferation of 7402-DEP, HepG2-DEP cells and control cells were examined by colony formation assay. **Figure S3.** (A) Representative phase contrast images of HepG2-DEP cells and their control cells. (B) IF for DEPTOR was shown in HLF-shDEP1/2 cells and their control cells. Scale bar: 30 μm. (C) Overexpression of snail expression promoted EMT in HLF-shDEP1 cells. (D) The transwell assay was used to detect the capacity of migration and invasion in the indicated cells following snail overexpression. (E) Representative images of IHC staining with anti-DEPTOR and anti-E-cadherin. The expression of DEPTOR was inversely correlated with that of E-cadherin. Scale bar: 300 μm (left panel) and 30 μm (right panel). The data represent means ± SEM from three independent experiments. *P < 0.05, **P < 0.01, ***P < 0.001. **Figure S4.** The sequences of a series of truncated or mutant DEPTOR 5′-promoter luciferase constructs. (DOCX 2973 kb)

## Abbreviations

CCK8: Cell Counting Kit-8
DEPTOR: DEP domain containing mTOR interacting protein
DFS: Disease-free survival
EMT: Epithelial-to-mesenchymal transition
H&E: Hematoxylin and eosin
HCC: Hepatocellular carcinoma
IF: Immunofluorescence
IHC: Immunohistochemistry
OD: Optical density
OS: Overall survival
PCR: Polymerase chain reaction
