# Supplementary figures and images for: DEPTOR induces a partial epithelial-to-mesenchymal transition and metastasis via autocrine TGFβ1 signaling and is associated with poor prognosis in hepatocellular carcinoma
Source: J Exp Clin Cancer Res. 2019 Jun 22;38:273. doi: 10.1186/s13046-019-1220-1 (PMC6588925; doi:10.1186/s13046-019-1220-1)

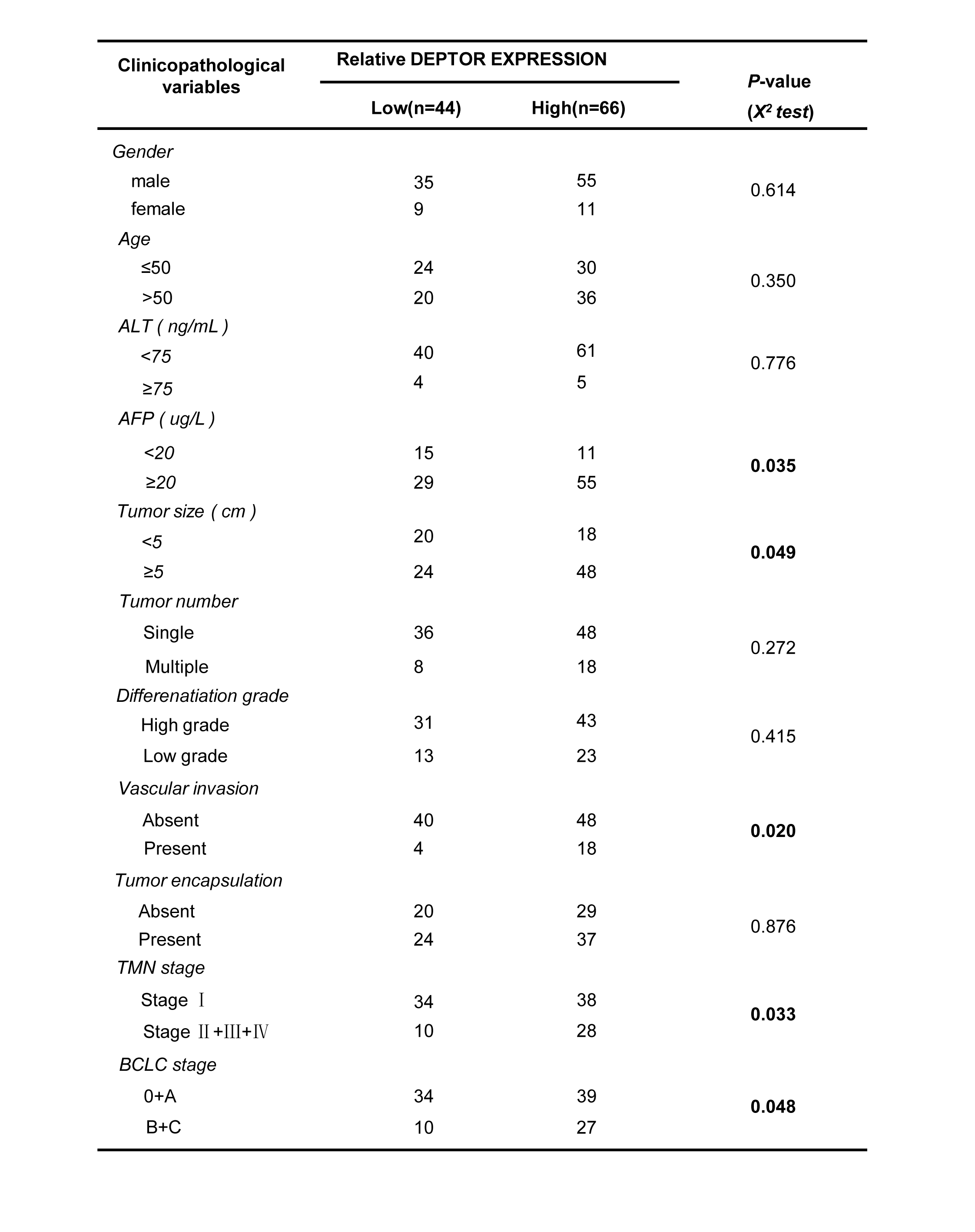

Supplement: Supplementary file 1 — Association of DEPTOR expression with Clinicopathologic Features in 110 Primary HCCs. (TIF 225 kb) [file 13046_2019_1220_MOESM1_ESM.tif]

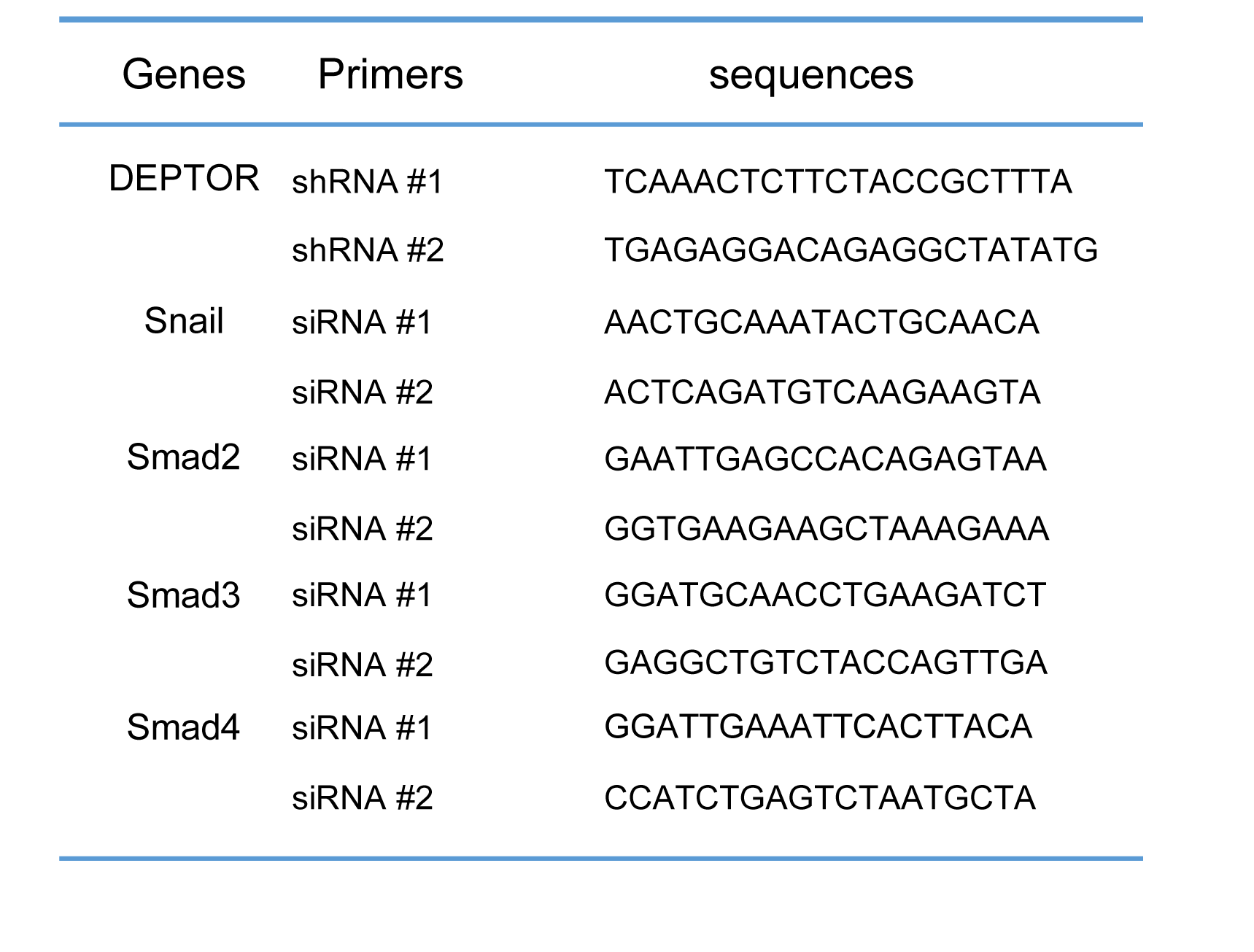

Supplement: Supplementary file 2 — The target sequences of shRNA and siRNA are listed in the table. (TIF 156 kb) [file 13046_2019_1220_MOESM2_ESM.tif]

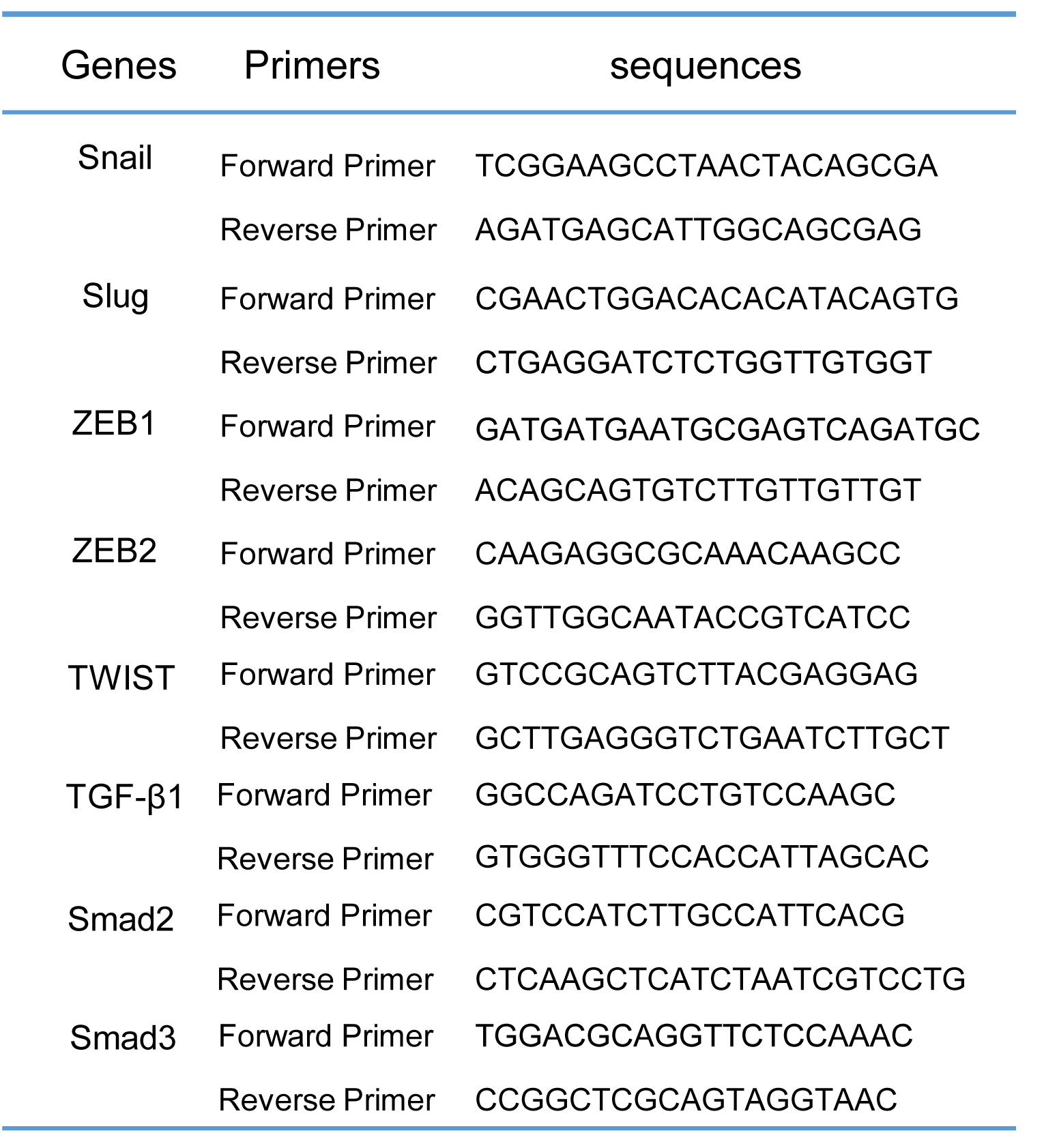

Supplement: Supplementary file 3 — The indicated primers used in the study were showed in the table. (TIF 241 kb) [file 13046_2019_1220_MOESM3_ESM.tif]
