# Supplementary material for: DEPTOR induces a partial epithelial-to-mesenchymal transition and metastasis via autocrine TGFβ1 signaling and is associated with poor prognosis in hepatocellular carcinoma
Source: J Exp Clin Cancer Res. 2019 Jun 22;38:273. doi: 10.1186/s13046-019-1220-1 (PMC6588925; doi:10.1186/s13046-019-1220-1)

**Materials and Method**

*Western blot analysis*

Western blot was performed as described previously[^1^](#_ENREF_1). Primary antibodies and their sources were as follows: DEPTOR (NBP1-49674, Novus Biologicals), E-cadherin (610181; BD Biosciences, San Jose, CA, USA), Occludin ([13409-1-AP,](http://www.ptgcn.com/products/OCLN-Antibody-13409-1-AP.htm) Proteintech), ZEB1 (21544-1-AP, Proteintech), ZEB2 (14026-1-AP, Proteintech), Twist (25465-1-AP, Proteintech), GAPDH (KC-5G4; KangChen Bio-tech, Inc., Shanghai, China). The following antibodies were purchased from Cell Signaling Technology, Inc. (Beverly, MA, USA): Snail (#3879), Slug (#9585), Smad2 (#5339), phospho-Smad2 (#18338), Smad3 (#9523), phosphor-Smad3 (#9520), P70S6K (#2708), phosphor-P70S6K (#9234), TGF-β antibody (#3711), β-actin (#4970).

*Cell viability Assays*

Cell viability was determined by using the Cell Counting Kit-8 (Beyotime Institute of Biotechnology) according to the manufacturer’s instructions. Indicated cells (counted by Cellometer Mini, Nexcelom Bioscience, Massachusetts, USA) were seeded in 96-well plates for 5 days with replacement of the culture media every 2 days. 100ul Cell Counting Kit-8 solution was added to the wells of the plates for 1 h to test the optical density (OD) value at 450 nm (Elx 800; BioTek Instruments, Inc., Winooski, VT, USA). For the colony formation assay, indicated cells were plated in 6-well plates and media were replaced with fresh culture media every 2 days. After 14 days, the plates were fixed with 4% formaldehyde and stained with 1% crystal violet (Sigma-Aldrich, USA) and photographed. Colonies were counted and analyzed using Alpha Innotech Imaging system (Alphatron Asia PteLtd, Singapore).

*Transwell assays and ELISA*

The transwell migration and invasion assay was performed as described previously[^2^](#_ENREF_2)^,^[^3^](#_ENREF_3). Each experiment was repeated three times. The ELISA assay was performed as described previously[^2^](#_ENREF_2). 3*10^5^ HCC cells were seeded into each well of 6-well plates and incubated for 24 h in 2ml serum free medium. Equal extracellular media was collected, activated by diluted hydrochloric acid and used for a TGF-β1 assay using the human/mouse TGF-β1 ELISA Ready-SET-Go! (2nd generation) kits (88-8350, eBioscience, San Diego, CA, USA) according to the manufacturer’s instructions

*Real-time PCR*

Real-time PCR was performed as described previously [^4^](#_ENREF_4). The following primers were got from primer bank. The Ct values of indicated genes were equilibrated to those of the internal control GAPDH. Relative expression was calculated using the 2-ΔΔCt method. Each experiment was repeated three times. The indicated primers used in the study were showed in supplemental Table 3.

References:

1 Chen, J. *et al.* 18beta-Glycyrrhetinic-acid-mediated unfolded protein response induces autophagy and apoptosis in hepatocellular carcinoma. *Scientific reports* **8**, 9365, doi:10.1038/s41598-018-27142-5 (2018).

2 Ding, Z. Y. *et al.* Reduced expression of transcriptional intermediary factor 1 gamma promotes metastasis and indicates poor prognosis of hepatocellular carcinoma. *Hepatology (Baltimore, Md.)* **60**, 1620-1636, doi:10.1002/hep.27273 (2014).

3 Chen, W. X. *et al.* MicroRNA-630 suppresses tumor metastasis through the TGF-beta- miR-630-Slug signaling pathway and correlates inversely with poor prognosis in hepatocellular carcinoma. *Oncotarget* **7**, 22674-22686, doi:10.18632/oncotarget.8047 (2016).

4 Chen, L. *et al.* Activin A induces growth arrest through a SMAD- dependent pathway in hepatic progenitor cells. *Cell Commun Signal.* **12**, 1-14 (2014).

Figure legend

Supplementary Fig 1: Western blotting analysis of relative DEPTOR expression in 53 HCC tissues (T) and its adjacent non-tumor tissues (N).

Supplementary Fig 2 (A) Western blotting was used to detect the overexpression efficiency of 7402 and HepG2 cells. (B) Proliferation of 7402-DEP, HepG2-DEP cells and control cells were examined by CCK8 assay. (C) Proliferation of 7402-DEP, HepG2-DEP cells and control cells were examined by colony formation assay.

Supplementary Fig 3 (A) Representative phase contrast images of HepG2-DEP cells and their control cells. (B) IF for DEPTOR was shown in HLF-shDEP1/2 cells and their control cells. Scale bar: 30µm. (C) Overexpression of snail expression promoted EMT in HLF-shDEP1 cells. (D) The transwell assay was used to detect the capacity of migration and invasion in the indicated cells following snail overexpression. (E) Representative images of IHC staining with anti-DEPTOR and anti-E-cadherin. The expression of DEPTOR was inversely correlated with that of E-cadherin. Scale bar: 300µm (left panel) and 30µm (right panel). The data represent means ± SEM from three independent experiments. **P*<0.05, ***P*<0.01, ****P*<0.001

Supplementary Fig 4 The sequences of a series of truncated or mutant DEPTOR 5'-promoter luciferase constructs.

Supplementary Fig 1


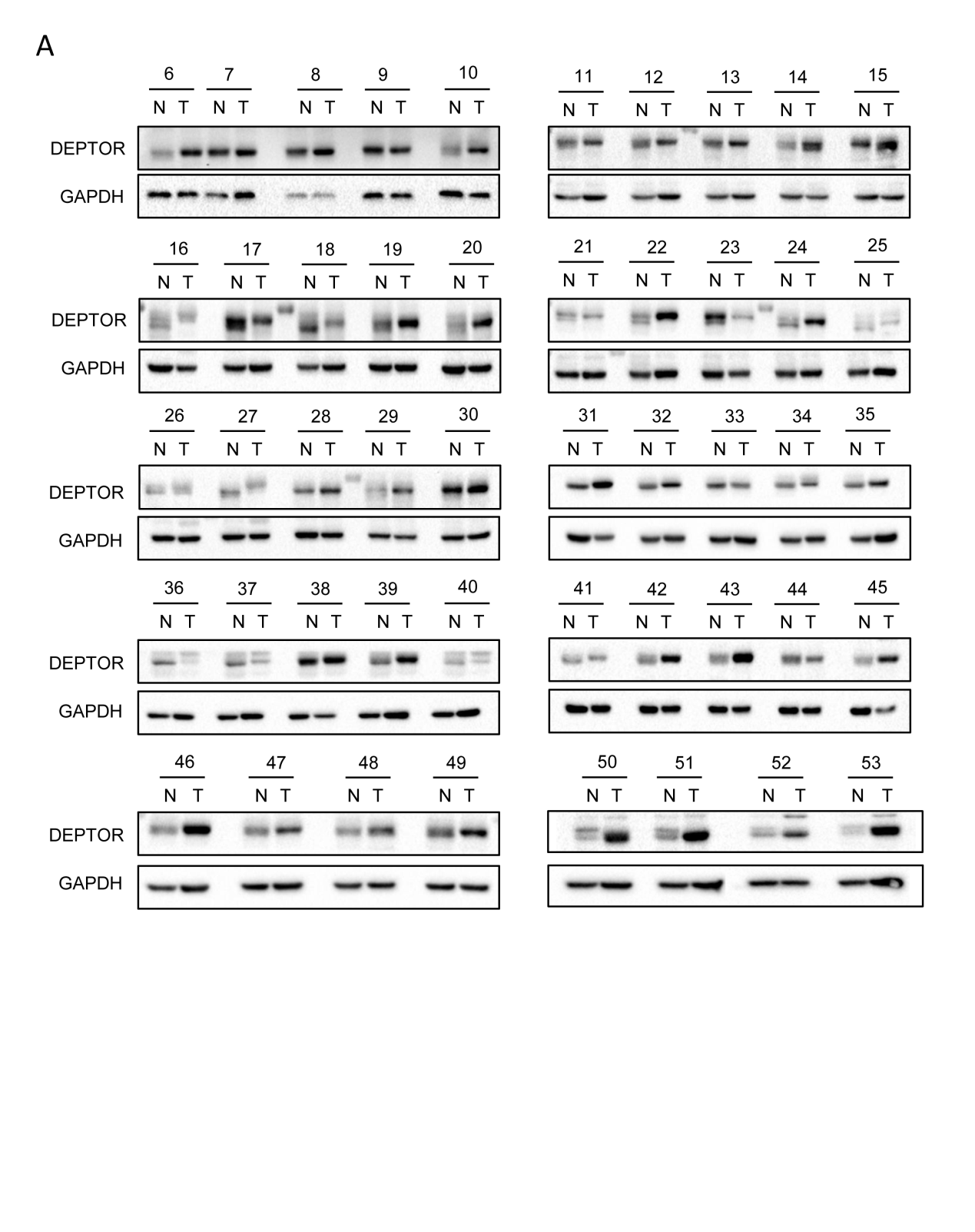


Supplementary Fig 2


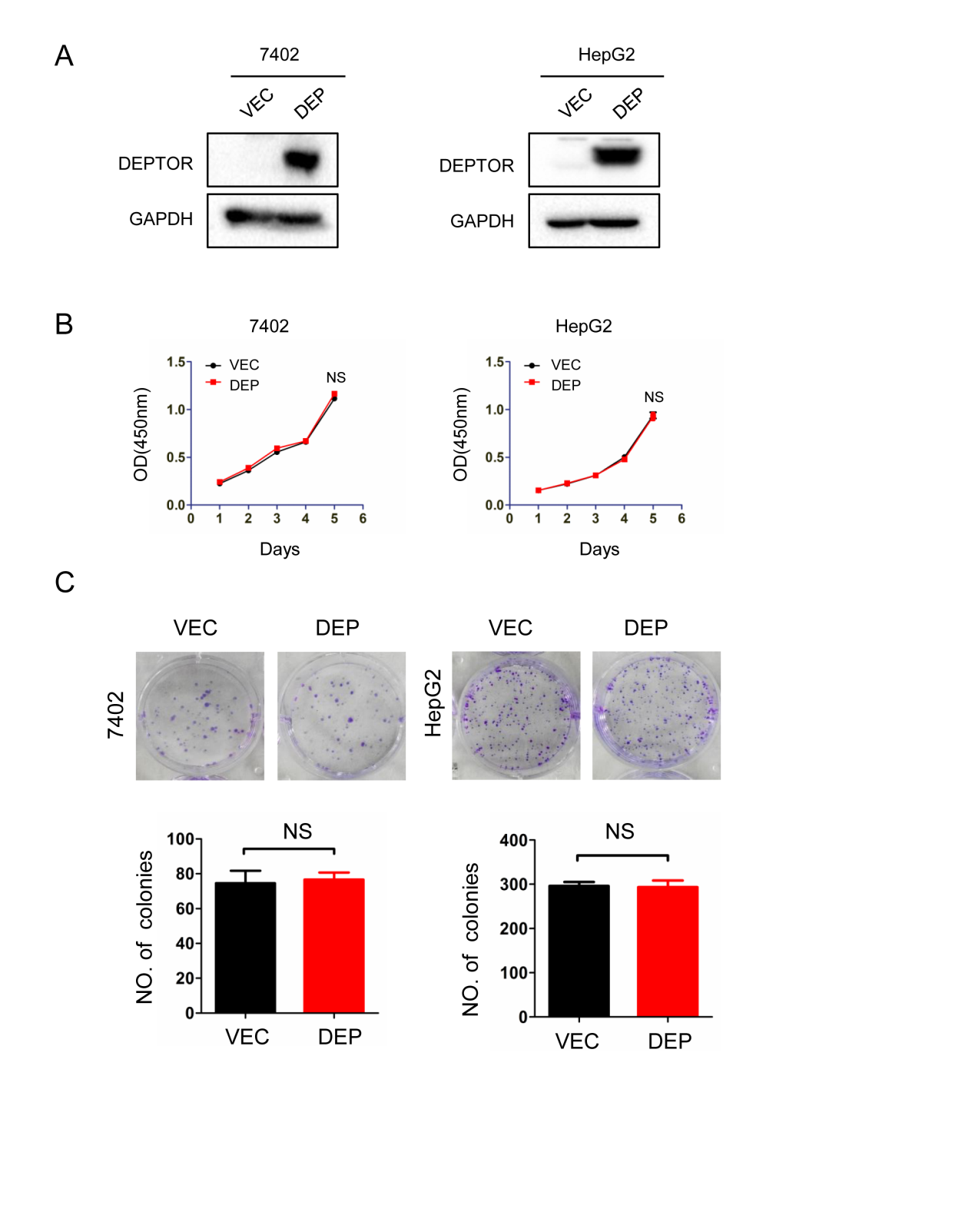


Supplementary Fig 3


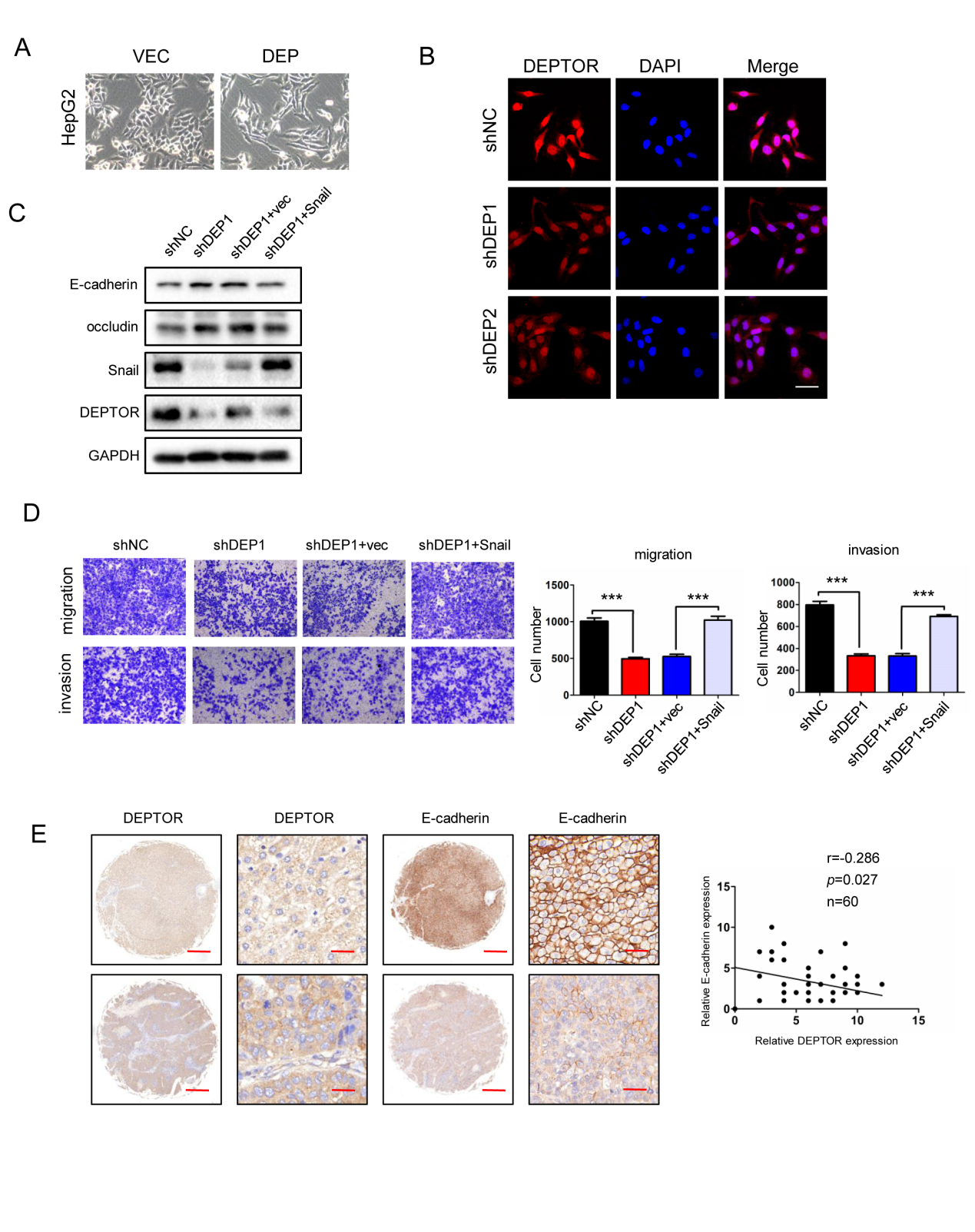


Supplementary Fig 4


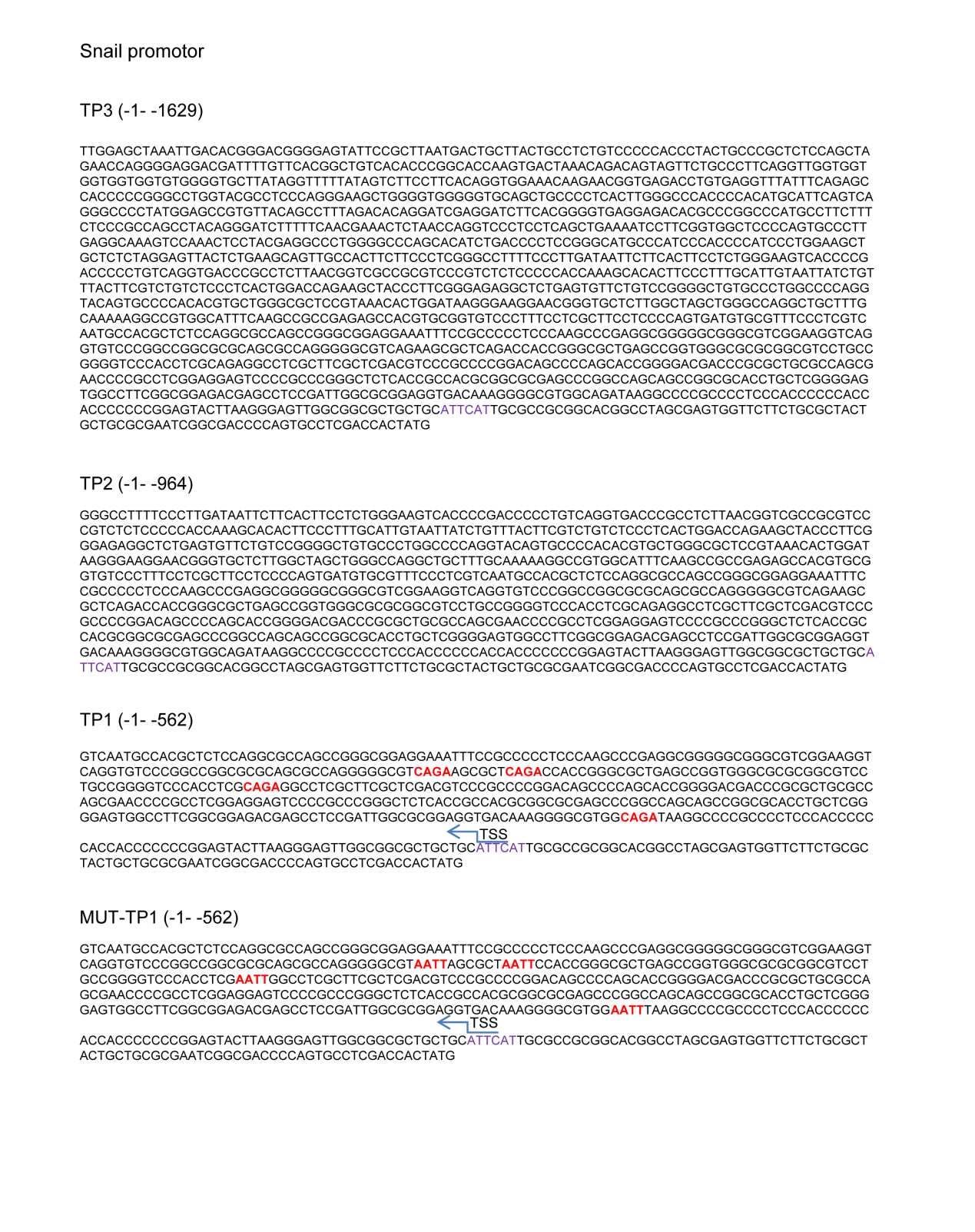

Supplement: Supplementary file 4 — Figure S1. Western blotting analysis of relative DEPTOR expression in 53 HCC tissues (T) and its adjacent non-tumor tissues (N). Figure S2. (A) Western blotting was used to detect the overexpression efficiency of 7402 and HepG2 cells. (B) Proliferation of 7402-DEP, HepG2-DEP cells and control cells were examined by CCK8 assay. (C) Proliferation of 7402-DEP, HepG2-DEP cells and control cells were examined by colony formation assay. Figure S3. (A) Representative phase contrast images of HepG2-DEP cells and their control cells. (B) IF for DEPTOR was shown in HLF-shDEP1/2 cells and their control cells. Scale bar: 30 μm. (C) Overexpression of snail expression promoted EMT in HLF-shDEP1 cells. (D) The transwell assay was used to detect the capacity of migration and invasion in the indicated cells following snail overexpression. (E) Representative images of IHC staining with anti-DEPTOR and anti-E-cadherin. The expression of DEPTOR was inversely correlated with that of E-cadherin. Scale bar: 300 μm (left panel) and 30 μm (right panel). The data represent means ± SEM from three independent experiments. *P < 0.05, **P < 0.01, ***P < 0.001. Figure S4. The sequences of a series of truncated or mutant DEPTOR 5′-promoter luciferase constructs. (DOCX 2973 kb) [file 13046_2019_1220_MOESM4_ESM.docx]
